# 3D-Cobalt-Dicyanamide-Derived 2D-Layered-Co(OH)_2_-Based Catalyst for Light-Driven Hydrogen Evolution

**DOI:** 10.1021/acsomega.4c00217

**Published:** 2024-02-09

**Authors:** Sina Sadigh Akbari, Ferdi Karadas

**Affiliations:** † Department of Chemistry, Faculty of Science, Bilkent University, 06800 Ankara, Turkey; ‡ UNAM—National Nanotechnology Research Center, Institute of Materials Science and Nanotechnology, Bilkent University, 06800 Ankara, Turkey

## Abstract

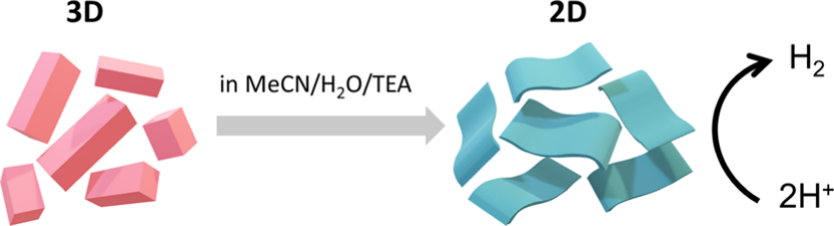

Derivation of 3D coordination polymers to produce active catalysts has been a feasible strategy to achieve a precise coordination sphere for the catalytic site. This study demonstrates the partial conversion of a 3D cobalt dicyanamide coordination polymer, Co-dca, to a 2D layered hydroxide–oxyhydroxide structure under photocatalytic conditions. The catalyst exhibits an activity as high as 28.3 mmol h^–1^ g^–1^ in the presence of a [Ru(bpy)_3_]^2+^/triethylamine (TEA) couple to maintain it for at least 12 h. Photocatalytic and characterization studies reveal that the dicyanamide ligand within the coordination polymer is crucial for governing modification and achieving a superior H_2_ evolution rate. Moreover, we observed the critical role of TEA as the hydrolyzing agent for the transformation process. This study displays that the metal dicyanamides can be utilized as templates for preparing active and robust catalysts.

## Introduction

Large-scale hydrogen generation from renewable sunlight employing a catalytic reaction is an ideal approach for sustainable energy production.^1,2^ Photocatalytic water splitting is one of the methods for H_2_ production, which utilizes solar energy cost-effectively and environmentally safely.^3,4^ Since the discovery of light-driven photoelectrochemical water splitting using TiO_2_ as a photocatalyst by Fujishima and Honda in 1972,^5^ many efforts have been made to develop efficient catalysts for hydrogen evolution reaction (HER).^6−8^

The majority of HER photocatalytic systems consist of a catalyst, a photosensitizer (PS), and a sacrificial electron donor. Various PSs have been developed, including organic and organometallic chromophores, which serve as both a light absorber and an electron carrier for the catalyst.^7,9−11^ Among them, ruthenium PSs have commonly been used for visible-light-driven HER studies due to their unique redox and photochemical properties.^12−21^ While metallic colloidal Pt or Pt-containing catalysts display excellent HER activity, they are rare and expensive.^22,23^ Earth-abundant metals such as cobalt, nickel, iron, and copper have gained significant attraction as potential HER catalysts over the past decades.^6,24,25^

Cobalt hydroxide, Co(OH)_2_, holds significant promise as a transition metal hydroxide in electrocatalytic and photocatalytic hydrogen evolution applications due to its distinctive multidimensional structure, capable of adopting both 2D and 3D morphologies.^26−28^ Fabrication of Co(OH)_2_ from various templates and precursors has been employed to construct a structure with a large surface area and enhanced charge transfer. For example, Wang et al. prepared porous Co(OH)_2_ materials from Co–MOF as a template, which exhibited superior energy storage performance for supercapacitors.^29^ Yang et al. utilized a simple method to synthesize mesoporous Co(OH)_2_ nanocubes derived from a Co–Co Prussian blue analogue (PBA).^30^ Since Co(OH)_2_ exhibits poor light harvesting efficiency and charge transfer capability, the typical strategy is to couple Co(OH)_2_ with suitable semiconductors or PSs for the light-driven HER process.^28,31−34^ In harsh alkaline environments, most cobalt-based catalysts transform into compounds including hydroxide–oxyhydroxide structures. This conversion could be restricted to the outer layer of the catalyst’s particles, forming an amorphous shell or a total transformation into amorphous structures.^35,36^

The coordination polymers containing a dicyanamide (dca) ligand, [N(CN)_2_]^−^, have attracted much attention due to their unconventional structures and magnetic properties.^37,38^ Unlike the well-known cyanide ligand, which only accepts one coordination mode, the dca ligand adopts various coordination modes using its nitrogen atoms.^39,40^ The cobalt dicyanamide structure, Co(dca)_2_, comprises a 3D network of octahedral metal ions connected by dca bridging groups. Electronic communication can be created between metal ions via a relatively short dicyanamide ligand, facilitating electron transfer for catalytic reactions. We recently demonstrated that Co(dca)_2_ coordination compound can be used as a selective photocatalyst for CO_2_ reduction in the presence of a ruthenium PS.^41^ Besides, adding triethanolamine as a sacrificial electron donor creates a basic reaction environment, leading to the decomposition of cobalt dicyanamide. Nevertheless, it is essential to note that this decomposition may improve the catalytic features by adjusting the experimental conditions. Therefore, Co(dca)_2_-derived Co(OH)_2_ structures could provide a facile pathway for developing efficient and stable HER catalysts.

Herein, we report the formation of a Co(OH)_2_ system derived from a Co(dca)_2_ coordination compound and its photocatalytic HER performance in the presence of [Ru(bpy)_3_]^2+^ as the light-harvesting component and triethylamine (TEA) as the sacrificial electron donor. The effect of various reaction parameters on the photocatalytic activity was explored and optimized. We observe that TEA serves as a hydrolyzing agent to partially transform cobalt dicyanamide into hydroxide–oxyhydroxide structures under photocatalytic experimental conditions. Our results reveal that the presence of the dicyanamide moiety contributes significantly to the enhanced performance of the catalyst during HER. The catalyst also exhibits a notable morphological evolution from a 3D cubic structure to a 2D layered one. We present a comprehensive characterization study to elucidate the role of experimental parameters in the conversion process and the photocatalytic activity.

## Experimental Section

### Chemicals and Reagents

All chemicals were used as-obtained without further purification. Cobalt(II) nitrate hexahydrate (Co(NO_3_)_2_·6H_2_O, Alfa Aesar, 99.9%), sodium dicyanamide (NaN(CN)_2_, Acros Organics, 97%), tris(2,2′-bipyridine)ruthenium(II) hexafluorophosphate ([Ru(bpy)_3_](PF_6_)_2_, Sigma-Aldrich, 98%), acetonitrile (MeCN, Sigma-Aldrich, >99.9%), *N*,*N*-dimethylformamide (DMF, Sigma-Aldrich, 99%), TEA (Carlo Erba reagents, 99.5%), triethanolamine (TEOA, Acros Organics, 97%) ascorbic acid (AA, Carlo Erba reagents, 99.0%). Deionized water (resistivity: 18 MΩ/cm) was used in all experiments.

### Synthesis of Co(dca)_2_

For the preparation of Co(dca)_2_, Co-dca, 9 mmol of NaN(CN)_2_ was dissolved in 4 mL of deionized water, added dropwise to a 4 mL solution containing 4.5 mmol of Co(NO_3_)_2_·6H_2_O, and stirred for 4 h at room temperature. A pink precipitate was collected by centrifugation, rinsed thoroughly with water, and finally dried at 70 °C.

### Preparation of Co-dca/TEA

To prepare Co-dca/TEA, 60 mg of as-synthesized Co-dca powder was dispersed in a MeCN/H_2_O/TEA mixed solution (7.6 mL/0.4 mL/2 mL) and vigorously stirred for 20 h at room temperature. Then, the obtained precipitate was centrifuged, washed with distilled water, and dried at 75 °C overnight.

### Photocatalytic HER Experiment

The H_2_ evolution studies were performed in a 16.5 mL round-bottomed flask capped with a rubber septum at room temperature. In a typical experiment, the reactor was filled with a mixed solution of 8 mL of MeCN/H_2_O (19/1; v/v) and 2 mL of TEA containing 8.6 mg of [Ru(bpy)_3_](PF_6_)_2_ (1 mM), and a certain amount of catalyst. The reaction solution was bubbled with N_2_ gas for 20 min before light illumination. The mixture was continuously stirred and irradiated under a 300 W Xe lamp (300 W; AM 1.5 global filter) with a 420 nm cutoff filter. At certain intervals, the headspace of the reactor was sampled by a gastight syringe and quantified by an Agilent 7829A gas chromatograph to determine the amount of evolved hydrogen gas. The gas chromatograph was equipped with a thermal conductivity detector, one split/splitless inlet (2:1 split ratio), and an Agilent 19095P-MS0 column (30 m × 530 μm × 25 μm HP–PLOT MoleSieve 5 A). Argon gas was the carrier gas and was used at a flow rate of 4 mL min^–1^. Standard H_2_/N_2_ gas mixtures with known concentrations were utilized to calibrate the gas chromatograph signal.

### Characterization

Powder X-ray diffraction (XRD) patterns of the samples were obtained on a Pananalytical X’PertPro multipurpose X-ray diffractometer using Cu K_α_ X-ray radiation (λ = 1.5405 Å) with a scan rate of 0.04 deg s^–1^ within the range of 10–70°. Fourier transform infrared (FT-IR) measurements were carried out by a Bruker Alpha Platinum-attenuated total reflectance (ATR) spectrometer with a diamond sample holder. The analyses were performed in transmittance mode with a resolution of 4 cm^–1^ and a wavenumber range of 400–4000 cm^–1^. X-ray photoelectron spectroscopy (XPS) studies were conducted with a Thermo Scientific K-Alpha X-ray photoelectron spectrometer system equipped with an Al K_α_ microfocused monochromator source (1486.6 eV) operating at 400 μm spot size and accompanied by a flood gun for charge neutralization. Powder samples were spread on a conducting copper tape for measurements. The morphology and chemical composition of the materials were studied by a scanning electron microscope (FEI–Quanta 200 FEG ESEM) with an energy-dispersive X-ray spectrometer (EDS) analyzer. Powder samples were placed on carbon tape for measurements. Transmission electron microscopy (TEM), scanning transmission electron microscopy, and high-angle annular dark field imaging were performed using an FEI Technai G2 F30 transmission electron microscope at 300 kV. For the sample preparation, powder samples were dispersed in ethanol and dropped on a carbon-coated copper grid. UV–vis absorption spectra were recorded on the Cary 5000 UV-A–vis–NIR spectrometer equipped with a Varian Cary 2500 internal diffuse reflectance accessory.

## Results and Discussion

**Figure 1:**
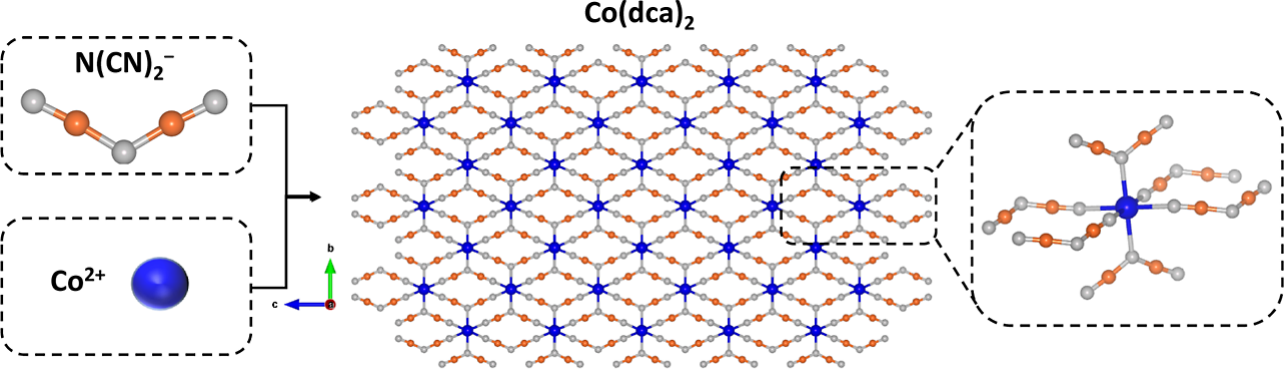
Schematic illustration showing the structure and synthesis procedure of Co(dca)_2_ (color code: Co = blue, C = orange, and N = gray).

Cobalt dicyanamide [Co(dca)_2_], Co-dca, was synthesized according to the procedures reported in the literature.^40^ The structure of Co-dca consists of a 3D network of octahedral metal ions bridged by dca groups with connectivity identical to that of the rutile polymorph of TiO_2_. The cobalt ions are coordinated to two axial dca ligands via their amide nitrogens and four equatorial dca ligands via the nitrile groups. Since the Co–N_amide_ bonds are longer than the Co–N_nitrile_, the octahedral sites are slightly elongated (Figure 1).^38,42^

**Figure 2:**
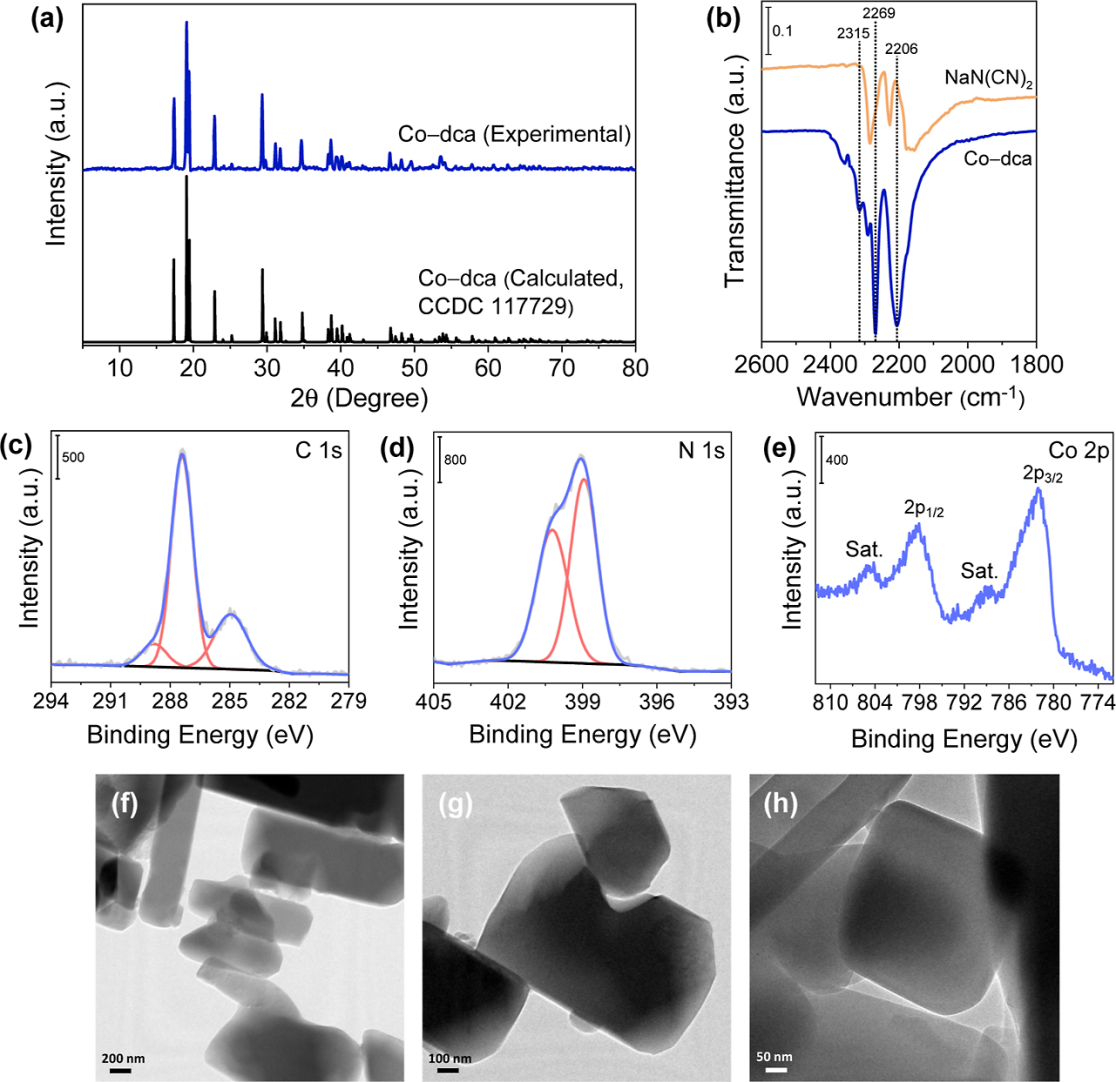
(a) Experimental and calculated (CCDC 117729) XRD patterns of Co-dca. (b) ATR–FTIR spectra of Co-dca sample and NaN(CN)_2_ between 1800 and 2600 cm^–1^. XPS spectra of (c) C 1s, (d) N 1s, and (e) Co 2p for pristine Co-dca. (f–h) TEM images of Co-dca.

The XRD pattern can be well indexed to that of Co(dca)_2_, which aligns with the findings of previously reported studies (CCDC 117729).^37,40^ The intense and sharp diffraction peaks verify the presence of a highly crystalline structure (Figure 2a). The FTIR spectrum of the sodium dicyanamide precursor exhibits three characteristic absorption bands at 2176, 2227, and 2284 cm^–1^, which can be attributed to the stretching modes of ν_s_(C≡≡N), ν_as_(C≡N), and ν_as_ + ν_s_(C≡N), respectively. For Co-dca, all three bands shift to higher wavenumbers (2206, 2269, and 2315 cm^–1^, respectively) due to the coordination of dca to Co sites (Figure 2b).^43−45^ Moreover, the absorption bands of the ν_s_(N–C) and ν_as_(N–C) stretching modes are observed at 964 and 1314 cm^–1^, respectively (Figure S1).^37,46^ The assignment of the other vibrations can be found in Table S1.

XPS analysis was performed to identify the chemical composition and valence state of Co-dca. The peaks of the C 1s spectrum located at 284.9, 287.4, and 288.8 eV can be assigned to the C atoms in the C–C bond (adventitious carbon species), the dicyanamide (N–C≡N), and the surface C=O groups, respectively (Figure 2c).^47,48^ The N 1s spectrum of Co-dca displays two peaks at 398.9 and 400.2 eV, which are associated with the nitrile nitrogen and the central amido nitrogen in the dicyanamide structure, respectively (Figure 2d).^49,50^ According to previous studies, the cobalt 2p_3/2_–2p_1/2_ spin–orbit splitting is close to 15 and 16 eV for diamagnetic Co^III^ and paramagnetic Co^II^ complexes, respectively.^51,52^ Furthermore, Co^II^ ions show broader 2p_3/2_ and 2p_1/2_ lines with more intense satellite peaks compared to those of Co^III^ ions.^52,53^ The Co 2p spectrum of Co-dca consists of broad 2p_3/2_ and 2p_1/2_ lines located at 782.1 and 798.2 eV, respectively, with a spin–orbit splitting of 16.1 eV. Another doublet at slightly higher binding energies corresponds to shakeup satellite peaks (Figure 2e). This suggests that most cobalt cations exist in +2 oxidation states.^52,54^ The morphological and structural properties of the sample were assessed further by scanning electron microscopy (SEM) and TEM analyses. TEM images indicate the cubic morphology of Co-dca particles of micrometer size (Figure 2f–h). The SEM–EDS spectrum confirms the presence of Co, C, and N elements with a 1:5.5:7.6 (Co/C/N) ratio, which is close to the stoichiometric ratio of Co-dca (Figure S2).

The photocatalytic hydrogen evolution ability of the catalyst was evaluated in the presence of ruthenium PS ([Ru(bpy)_3_](PF_6_)_2_) under visible light irradiation. [Ru(bpy)_3_]^2+^ complex absorbs visible light effectively due to the characteristic metal-to-ligand charge transfer (MLCT, absorption band centered at 451 nm), in which an electron from a d orbital of Ru is transferred to a π* orbital of one of the bpy ligands (Figure S3).^55^ We conducted several control experiments to select an optimal reaction medium for the efficient operation of the photocatalytic HER. First, the effect of the sacrificial electron donor on the catalytic activity was examined by employing common hole scavengers including TEA, triethanolamine (TEOA), and AA. TEA exhibits the highest hydrogen production rate, with around 2 and 17 times higher activity than that obtained for TEOA and AA, respectively. Moreover, the photocatalytic activity decreases drastically in all cases when water is used as the only solvent (Figure S4a). This decline may be interpreted by the complete dissolution of Co-dca in 100% (volume %) of the water solution. In the TEA and TEOA cases, replacing acetonitrile (MeCN) with DMF leads to a notable reduction in the catalytic activity, suggesting that MeCN is one of the key elements in the high H_2_ evolution process (Figure S4b). We selected TEA as the sacrificial electron donor in the MeCN/H_2_O solution to achieve a superior hydrogen production rate based on these results.

We also investigated the effect of the quantity of the catalyst on the HER catalytic performance (Figure S5). When 1 mg of Co-dca was introduced into the system, the H_2_ yield reached the maximum value of 65,100 μmol g^–1^ under 3 h irradiation. Note that an increase in the amount of catalyst leads to a decrease in the level of H_2_ production.

**Figure 3:**
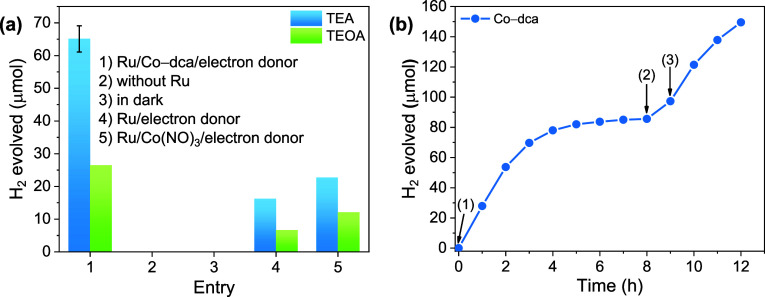
(a) Rate of H_2_ evolution with Co-dca (1 mg), [Ru(bpy)_3_]^2+^ (1 mM), and electron donor (2 mL; entry 1), without [Ru(bpy)_3_]^2+^ (entry 2), without light (entry 3), and without Co-dca (entry 4), when Co-dca is replaced with Co(NO_3_)_2_ as the catalyst (entry 5). The reactions are performed in the presence of TEA (blue bars) and TEOA (green bars) as the sacrificial electron donor under 3 h visible light irradiation. (b) Long-term H_2_ yield of Co-dca under visible light (λ > 420 nm) irradiation. (1) Starting conditions: 1 mg of catalyst, 8.6 mg of [Ru(bpy)_3_](PF_6_)_2_ (1 mM), 8 mL of MeCN/H_2_O (19/1, v/v), and 2 mL of TEA, (2) 8.6 mg of [Ru(bpy)_3_](PF_6_)_2_ (1 mM) added; (3) 2 mL of TEA added.

Various control studies were also carried out to explore the role of each component in the HER process (Figure 3a). When the photocatalytic reaction was operated using Ru PS, Co-dca, and TEA, a hydrogen generation rate of 65.1 μmol was achieved during the 3 h photocatalytic experiment (entry 1, the value is the mean of four repeated experiments with a corresponding error bar). H_2_ gas was not detected in the absence of the Ru PS or light, proving that the HER process depends on the excitation of the PS by light absorption (entries 2 and 3). If the catalyst is not added, the system yields a much lower activity (16.2 μmol of H_2_), verifying the critical role of Co-dca as the catalyst (entry 4). Co(NO_3_)_2_ was replaced with Co-dca to evaluate the effect of the dca ligand on the catalytic activity (entry 5). A hydrogen production rate (22.7 μmol) slightly higher than that in the no-catalyst case (entry 4) is obtained, suggesting that the activity of the cobalt site is enhanced when it is coordinated to dca groups. The same set of experiments were also performed with TEOA as the sacrificial electron donor reagent. While TEOA shows an identical feature to that of TEA, the amount of produced hydrogen is lower in all cases, indicating that TEA is a more efficient sacrificial electron donor for Co-dca/[Ru(bpy)_3_]^2+^ pair. The turnover frequency (TOF, the mole of evolved H_2_ per mole of the catalyst per time) is determined as 1.48 × 10^–3^ s^–1^ in the first h of the photocatalytic experiment (27.9 μmol of H_2_) for Co-dca. Note that all of the cobalt sites are assumed to be catalytically active to calculate the lower limit of the TOF value.

Studies of the reaction time reveal a linear relationship between the H_2_ yield and the irradiation time in the first 3 h of the photocatalytic experiment (Figure 3b). The maximum H_2_ production rate was achieved in the first h of the reaction, which was 27,900 μmol g^–1^ h^–1^. After this period, a decay in the hydrogen evolution was observed, which could be associated with the decomposition of Ru PS or consumption of the sacrificial electron donor.^21,56,57^ To elucidate the origin of the decay, an additional portion of Ru PS (1 mM) was added to the reaction solution (point 2). Then, after 1 h irradiation, 2 mL of fresh TEA was added at point 3. While recharging the solution only with Ru PS (8–9 h interval) restores 42% of activity, replenishing it with Ru PS and TEA (9–10 h interval) regenerates 86% of activity. The similarity of the photocatalytic performance after points 1 and 3 confirms that the activity of the catalyst is mostly retained.

**Figure 4:**
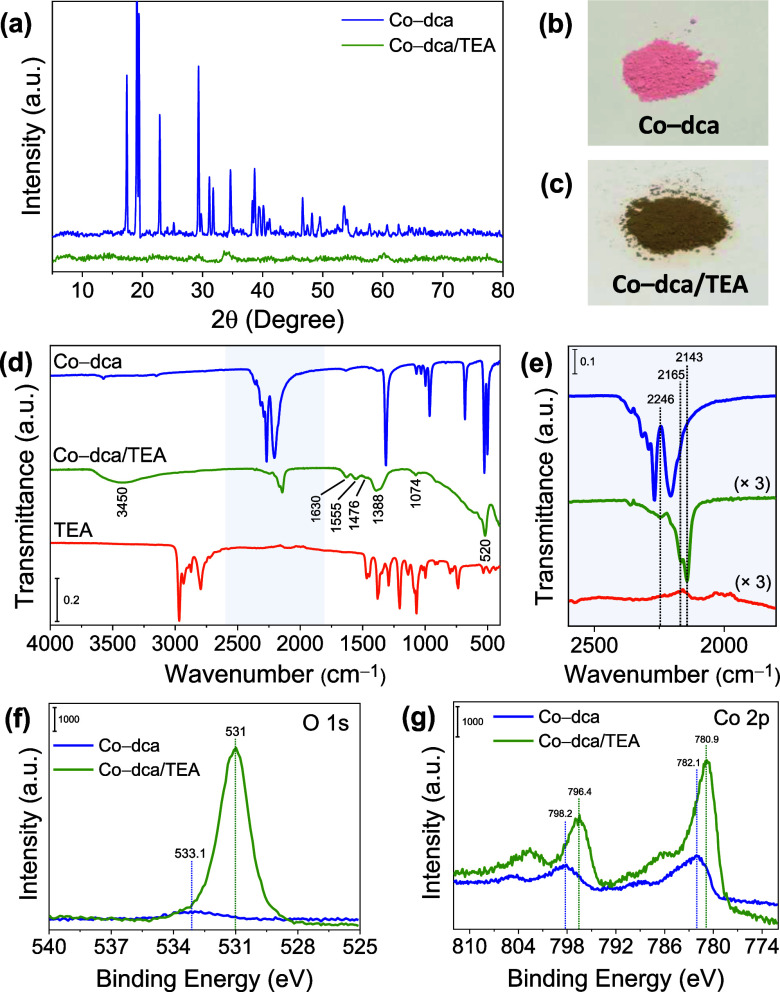
(a) XRD patterns of as-synthesized Co-dca and Co-dca in a MeCN/H_2_O/TEA solution (Co-dca/TEA). Photographs of (b) Co-dca and (c) Co-dca/TEA powders. ATR–FTIR spectra of Co-dca, Co-dca/TEA, and TEA between (d) 400–4000 cm^–1^ and (e) 1800–2600 cm^–1^. XPS spectra of (f) O 1s and (g) Co 2p for as-synthesized Co-dca and Co-dca/TEA.

We consistently observed a clear and noticeable change in the color of the catalyst throughout the photocatalytic experiments, suggesting that the catalyst undergoes chemical and structural transformations during the catalytic hydrogen evolution process. Our ongoing investigation aims to unravel the nature of this conversion as it can provide valuable insights into the influence of the photocatalytic reaction condition on the catalyst’s structure. Therefore, we performed a comprehensive characterization study on Co-dca after treating it with a MeCN/H_2_O/TEA solution (abbreviated as Co-dca/TEA; see the Experimental Section for details). The XRD pattern of Co-dca disappears completely, suggesting a transformation of the crystalline Co-dca structure to an amorphous phase (Figure 4a). The color of the Co-dca powder also changed notably from pink to dark green–gray after dispersion in the MeCN/H_2_O/TEA solution (Figure 4b,c). The irreversible color change observed in the catalyst serves as a visual indicator of the significant structural modifications. TEA is a highly basic tertiary alkylamine known not only as a reducing agent in photochemical reactions but also as a widely used precursor for the controlled synthesis of metal hydroxides and metal oxides.^58−60^ For example, TEA has recently been employed to synthesize Co(OH)_2_ nanosheets with CoOOH as the impurity.^61^ Given the possible role of TEA as a hydrolyzing agent, the formation of cobalt hydroxide and cobalt oxyhydroxide species is expected. Moreover, Indra et al. reported the transformation of PBAs, which are cyanide-based materials, into layered hydroxide–oxyhydroxide structures in the alkaline media during the water oxidation process.^35^ The broad absorption band at ca. 3450 cm^–1^ in the FTIR spectrum of Co-dca/TEA could be ascribed to the O–H stretching vibration of interlayer water molecules and hydrogen-bound OH groups (Figure 4d). The observed band at 1630 cm^–1^ is associated with the bending mode of water molecules.^62,63^ The bands located at 1555, 1476, 1388, and 1074 cm^–1^ correspond to the vibration modes of carbonate groups from carbon dioxide present in the air.^64,65^ While absorptions below 1000 cm^–1^ are attributed to Co–O stretching and Co–OH bending vibrations,^64,66^ and the band at 520 cm^–1^ can be assigned to γ(N–C≡N) vibration of dicyanamide. Cyanide stretch modes shift to lower wavenumbers compared with that of pristine Co-dca, verifying a variation in the coordination environment of cobalt ions (Figure 4e). The characteristic bands of TEA, including the stretching vibration of saturated C–H bonds (2800–3000 cm^–1^) and the bending vibration of N–H bonds (900–1400 cm^–1^), are not observed in the FTIR spectrum of Co-dca/TEA,^67^ suggesting that TEA is not present in the structure. Infrared studies thus indicate the presence of dca, hydroxyl, and water molecules in Co-dca/TEA.

XPS measurements were further carried out to identify the chemical composition of Co-dca/TEA. The presence of the Co(OH)_2_ phase in Co-dca/TEA is evident from the O 1s spectrum, which reveals an intense hydroxide peak at ca. 531 eV, while Co-dca shows a small peak at 533.1 eV, arising from the C=O species on the surface (Figure 4f).^65,68^ The spectra correlate well with the SEM–EDS analysis result of Co-dca/TEA, which displays a significant increase in the quantity of the elemental O compared to that in Co-dca (Figure S6). A distinct change in the oxidation state and electronic environment of cobalt has been recorded by the XPS studies. While the Co 2p spectrum of Co-dca reveals that the majority of cobalt ions are in +2 oxidation states, the narrower Co 2p_3/2_ and 2p_1/2_ lines and lower spin–orbit splitting energy difference compared to those of Co-dca verify that the cobalt cations are partially oxidized to Co^3+^ in Co-dca/TEA (Figure 4g). This may be due to the formation of the CoOOH phase in addition to the Co(OH)_2_ phase, resulting in the oxidation of cobalt ions to +3.

**Figure 5:**
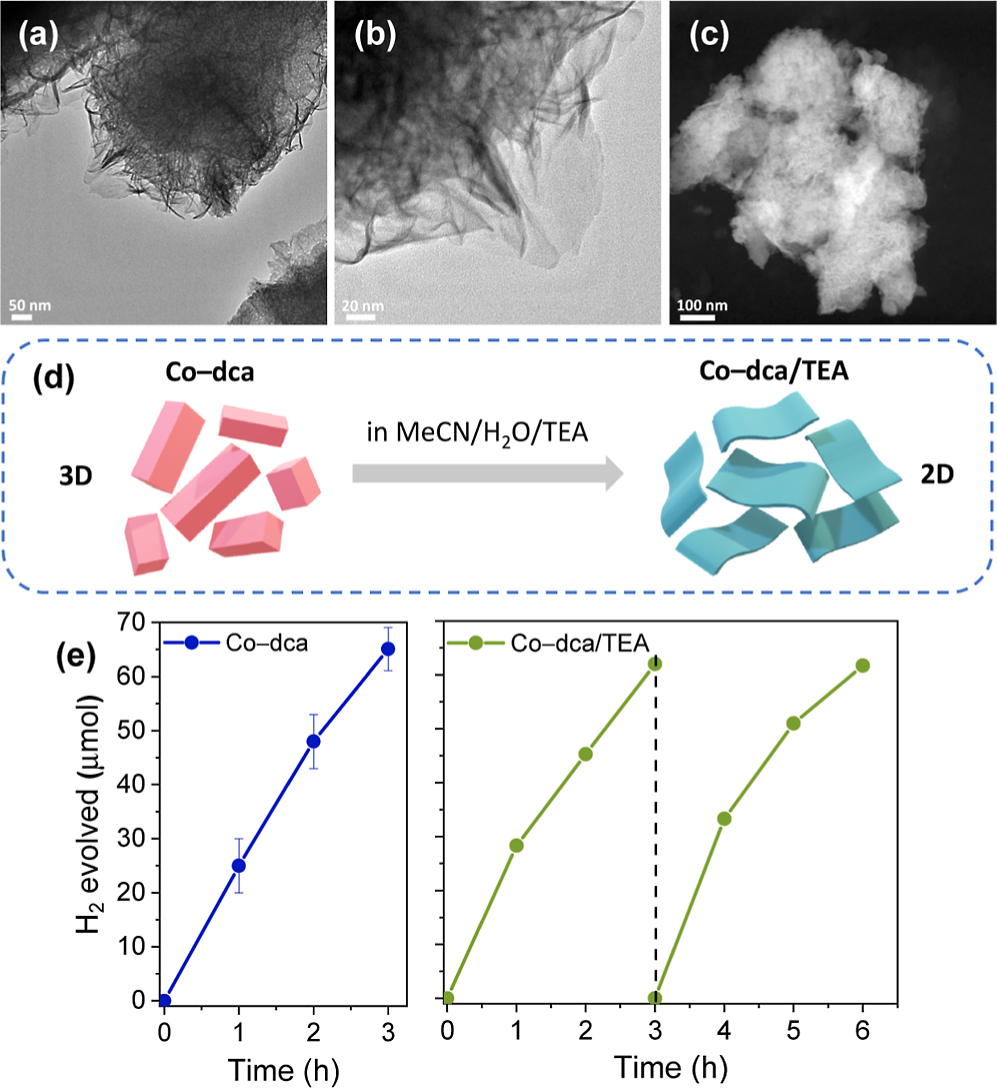
(a,b) TEM and (c) HAADF–STEM images of Co-dca/TEA. (d) Schematic representation reveals the morphological variation of Co-dca in a MeCN/H_2_O/TEA solution. (e) Time courses of hydrogen evolution for as-synthesized Co-dca and Co-dca/TEA (the catalyst was dispersed in a fresh solution containing Ru PS in the 2nd cycle). Reaction conditions: 1 mg of catalyst, 1 mM of [Ru(bpy)_3_](PF_6_)_2_, 8 mL MeCN/H_2_O (19/1, v/v), and 2 mL of TEA under visible light irradiation (λ > 420 nm).

TEM images (Figures 5a–c and S7) depict that Co-dca/TEA possesses a typical layered-like morphology with a relatively smaller particle size than that of as-synthesized Co-dca. Overall, the characterization studies indicate that Co-dca is converted to an amorphous Co(OH)_2_ phase (or a combination of Co(OH)_2_ and CoOOH phases) in the presence of the MeCN/H_2_O/TEA solution, which not only alters the coordination environment of cobalt cations but also transforms the 3D network of Co-dca into a 2D layered structure, providing more active sites for the HER process (Figure 5d). Therefore, the structure of the catalyst could be described as an amorphous 2D Co(OH)_2_/CoOOH structure on a 3D Co-dca platform. However, the total conversion of the material is not detected due to the presence of the cyanide stretching mode in the infrared spectrum. Our future research efforts are dedicated to elucidating the specific mechanisms governing this transformation in order to achieve a more comprehensive understanding of its catalytic effects.

Both pristine Co-dca and Co-dca/TEA were investigated under identical experimental conditions (Figure 5e). Co-dca/TEA maintained its photocatalytic HER activity for at least two consequent cycles, which is comparable to that of Co-dca. Although the structure and morphology of Co-dca are altered in the presence of a MeCN/H_2_O/TEA solution, the hydrogen evolution activity remains unaffected. Photocatalytic activities of previously reported catalysts (listed in Table S2) reveal that Co-dca/TEA has remarkable activity compared to that of systems containing Co(OH)_2_ as the HER catalyst. Our results, thus, indicate that the coordination of cobalt sites to dca groups prior to the transformation process is crucial for high photocatalytic activity. Note that Co(NO_3_)_2_ exhibited a significantly lower H_2_ formation yield than that of Co-dca.

**Figure 6:**
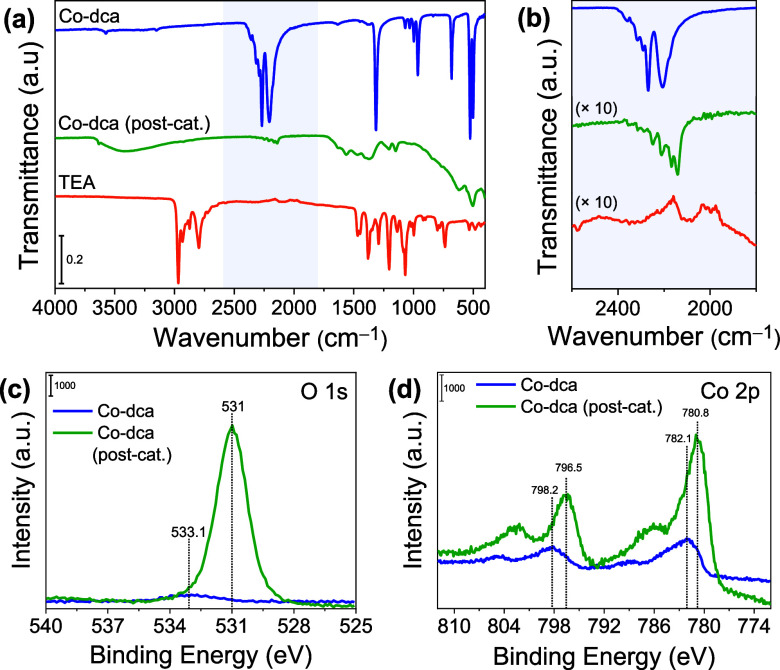
ATR–FTIR spectra of pristine Co-dca, postcatalytic Co-dca, and TEA between (a) 400–4000 cm^–1^ and (b) 1800–2600 cm^–1^. XPS spectra of (c) O 1 s and (d) Co 2p for pristine Co-dca and postcatalytic Co-dca sample.

The structural features of the postcatalytic Co-dca sample after the 12 h HER experiment were compared to those of Co-dca/TEA by employing various characterization methods. The FTIR spectra of Co-dca (postcatalytic) and Co-dca/TEA are similar, indicating that only a fraction of the Co-dca converted to Co(OH)_2_ during the photocatalytic process (Figure 6a,b). The O 1s and Co 2p XPS spectra of the postcatalytic sample exhibit similar profiles to those of Co-dca/TEA, verifying the formation of hydroxide–oxyhydroxide structures (Figure 6c,d). Moreover, the morphological changes in Co-dca were also examined using TEM analysis (Figure S8). While Co-dca initially displays a 3D coordination network, it transforms into a 2D layered structure at the end of the 3 h photocatalytic reaction, which is analogous to the morphology of Co-dca/TEA. Thus, the cobalt dicyanamide coordination polymer undergoes rapid structural and morphological modifications even during a 3 h HER experiment in a MeCN/H_2_O/TEA solution.

**Figure 7:**
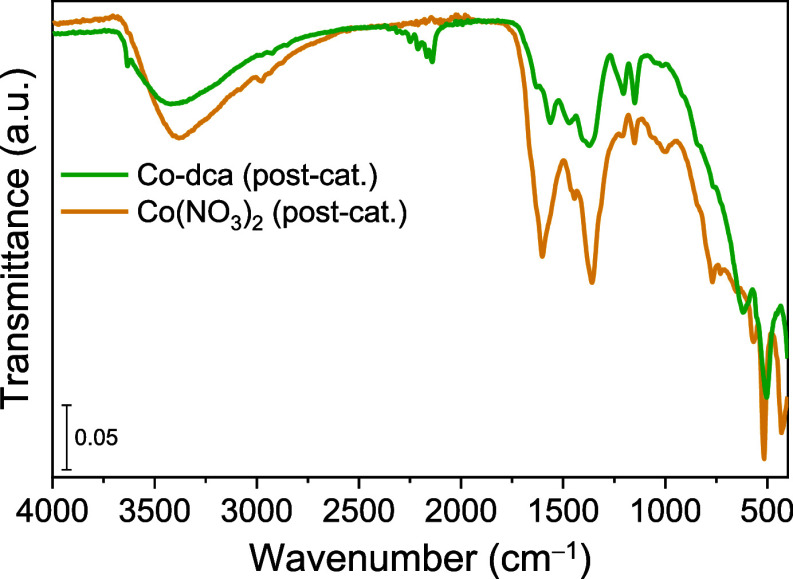
ATR–FTIR spectra of postcatalytic Co-dca and Co(NO_3_)_2_ samples.

Infrared spectroscopy was utilized to explore the effect of the solvent on the transformation of Co-dca (Figure S9). The FTIR spectra of Co-dca in MeCN and TEA are identical to the pure sample, revealing that Co-dca is stable in these media. However, FTIR profiles were altered when Co-dca was dispersed in MeCN/TEA and MeCN/H_2_O/TEA solutions. The infrared analysis also revealed that the addition of Co(NO_3_)_2_ powder to the reaction solution (MeCN/H_2_O/TEA) yielded cobalt hydroxide without a dca group in the postcatalytic sample (Figure 7). Despite the formation of cobalt hydroxide, the hydrogen generation rate was significantly reduced when Co(NO_3_)_2_ was used as the catalyst compared to that of Co-dca. These results suggest that enhanced photocatalytic performance requires not only structural modification but also the presence of dicyanamide. Thus, the distinct chemical structure and properties of dicyanamide must be present to obtain superior photocatalytic activity observed with Co-dca.

## Conclusions

A cobalt dicyanamide coordination compound, which consists of a 3D network of octahedral metal ions surrounded by dca groups, was prepared by a facile coprecipitation method. A series of photocatalytic studies under various reaction conditions were performed to reveal that the use of TEA as the sacrificial electron donor in the MeCN/H_2_O solution is crucial for achieving a high HER performance. Comprehensive studies indicate that Co-dca partially transforms to 2D-layered cobalt hydroxide–oxyhydroxide structures in a MeCN/H_2_O/TEA solution (the photocatalytic reaction condition) during HER. The Co-dca-derived catalyst exhibits a notable H_2_ yield of 27,900 μmol g^–1^ h^–1^ in the presence of Ru PS, which is robust for at least 12 h photocatalytic experiment. The experimental findings further reveal the critical role of dca groups in the high activity of the derived catalysts, indicating that cobalt dicyanamide provides an ideal platform for the facile synthesis of active cobalt hydroxide materials for light-driven hydrogen evolution. Dicyanamide presumably includes unique chemical features that boost the efficiency of the photocatalytic process. Without dca, the ability of the catalyst to convert light energy into the desired chemical products (i.e., H_2_) is significantly limited. While our present study has provided valuable insights into the catalytic process, the catalyst’s structure and composition and the reaction mechanism require a comprehensive investigation, which could be the focal point of a future study.

## Supporting Information Available

ATR–FTIR spectra, TEM images, SEM images, EDS analysis, UV–vis spectrum, and photocatalytic HER experiments (PDF)

## References

[ref1] Dutta S. Review on Solar Hydrogen: Its Prospects and Limitations. Energy Fuels 2021, 35, 11613–11639. 10.1021/acs.energyfuels.1c00823.

[ref2] Takata T.; Domen K. Particulate Photocatalysts for Water Splitting: Recent Advances and Future Prospects. ACS Energy Lett. 2019, 4, 542–549. 10.1021/acsenergylett.8b02209.

[ref3] Hisatomi T.; Domen K. Reaction Systems for Solar Hydrogen Production via Water Splitting with Particulate Semiconductor Photocatalysts. Nat. Catal. 2019, 2, 387–399. 10.1038/s41929-019-0242-6.

[ref4] Wang Q.; Domen K. Particulate Photocatalysts for Light-Driven Water Splitting: Mechanisms, Challenges, and Design Strategies. Chem. Rev. 2020, 120, 919–985. 10.1021/acs.chemrev.9b00201. 31393702

[ref5] Fujishima A.; Honda K. Electrochemical Photolysis of Water at a Semiconductor Electrode. Nature 1972, 238, 37–38. 10.1038/238037a0. 12635268

[ref6] Dalle K. E.; Warnan J.; Leung J. J.; Reuillard B.; Karmel I. S.; Reisner E. Electro- and Solar-Driven Fuel Synthesis with First Row Transition Metal Complexes. Chem. Rev. 2019, 119, 2752–2875. 10.1021/acs.chemrev.8b00392. 30767519 PMC6396143

[ref7] Willkomm J.; Orchard K. L.; Reynal A.; Pastor E.; Durrant J. R.; Reisner E. Dye-Sensitised Semiconductors Modified with Molecular Catalysts for Light-Driven H_2_ Production. Chem. Soc. Rev. 2016, 45, 9–23. 10.1039/C5CS00733J. 26584204

[ref8] Hisatomi T.; Kubota J.; Domen K. Recent Advances in Semiconductors for Photocatalytic and Photoelectrochemical Water Splitting. Chem. Soc. Rev. 2014, 43, 7520–7535. 10.1039/C3CS60378D. 24413305

[ref9] Lazarides T.; McCormick T.; Du P.; Luo G.; Lindley B.; Eisenberg R. Making Hydrogen from Water Using a Homogeneous System without Noble Metals. J. Am. Chem. Soc. 2009, 131, 9192–9194. 10.1021/ja903044n. 19566094

[ref10] Rupp M.; Auvray T.; Rousset E.; Mercier G. M.; Marvaud V.; Kurth D. G.; Hanan G. S. Photocatalytic Hydrogen Evolution Driven by a Heteroleptic Ruthenium(II) Bis(Terpyridine) Complex. Inorg. Chem. 2019, 58, 9127–9134. 10.1021/acs.inorgchem.9b00698. 31247814

[ref11] Andreiadis E. S.; Chavarot-Kerlidou M.; Fontecave M.; Artero V. Artificial Photosynthesis: From Molecular Catalysts for Light-Driven Water Splitting to Photoelectrochemical Cells. Photochem. Photobiol. 2011, 87, 946–964. 10.1111/j.1751-1097.2011.00966.x. 21740444

[ref12] Lv H.; Song J.; Zhu H.; Geletii Y. V.; Bacsa J.; Zhao C.; Lian T.; Musaev D. G.; Hill C. L. Visible-Light-Driven Hydrogen Evolution from Water Using a Noble-Metal-Free Polyoxometalate Catalyst. J. Catal. 2013, 307, 48–54. 10.1016/j.jcat.2013.06.028.

[ref13] Yuhas B. D.; Smeigh A. L.; Douvalis A. P.; Wasielewski M. R.; Kanatzidis M. G. Photocatalytic Hydrogen Evolution from FeMoS-Based Biomimetic Chalcogels. J. Am. Chem. Soc. 2012, 134, 10353–10356. 10.1021/ja303640s. 22662744

[ref14] Tsuji Y.; Yamamoto K.; Yamauchi K.; Sakai K. Near-Infrared Light-Driven Hydrogen Evolution from Water Using a Polypyridyl Triruthenium Photosensitizer. Angew. Chem., Int. Ed. 2018, 57, 208–212. 10.1002/anie.201708996. 29034550

[ref15] Kishimoto F.; Mochizuki D.; Maitani M. M.; Suzuki E.; Wada Y. Construction of Highly Hierarchical Layered Structure Consisting of Titanate Nanosheets, Tungstate Nanosheets, Ru(bpy)_3_^2+^, and Pt(terpy) for Vectorial Photoinduced Z-Scheme Electron Transfer. ACS Appl. Mater. Interfaces 2018, 10, 37150–37162. 10.1021/acsami.8b14749. 30280563

[ref16] Marguet S. C.; Stevenson M. J.; Shafaat H. S. Intramolecular Electron Transfer Governs Photoinduced Hydrogen Evolution by Nickel-Substituted Rubredoxin: Resolving Elementary Steps in Solar Fuel Generation. J. Phys. Chem. B 2019, 123, 9792–9800. 10.1021/acs.jpcb.9b08048. 31608640

[ref17] Wang P.; Liang G.; Smith N.; Hill K.; Donnadieu B.; Webster C. E.; Zhao X. Enhanced Hydrogen Evolution in Neutral Water Catalyzed by a Cobalt Complex with a Softer Polypyridyl Ligand. Angew. Chem., Int. Ed. 2020, 59, 12694–12697. 10.1002/anie.202002640. 32307871

[ref18] Aoi S.; Mase K.; Ohkubo K.; Fukuzumi S. Mechanism of a One-Photon Two-Electron Process in Photocatalytic Hydrogen Evolution from Ascorbic Acid with a Cobalt Chlorin Complex. Chem. Commun. 2015, 51, 15145–15148. 10.1039/C5CC05064B. 26323791

[ref19] Deshmukh M. S.; Mane V. S.; Kumbhar A. S.; Boomishankar R. Light-Driven Hydrogen Evolution from Water by a Tripodal Silane Based Co^II^_6_L_18_ Octahedral Cage. Inorg. Chem. 2017, 56, 13286–13292. 10.1021/acs.inorgchem.7b02074. 29043789

[ref20] Niu W.; Moehl T.; Cui W.; Wick-Joliat R.; Zhu L.; Tilley S. D. Extended Light Harvesting with Dual Cu_2_O-Based Photocathodes for High Efficiency Water Splitting. Adv. Energy Mater. 2018, 8, 1702323 10.1002/aenm.201702323.

[ref21] Chakraborty S.; Edwards E. H.; Kandemir B.; Bren K. L. Photochemical Hydrogen Evolution from Neutral Water with a Cobalt Metallopeptide Catalyst. Inorg. Chem. 2019, 58, 16402–16410. 10.1021/acs.inorgchem.9b02067. 31773947 PMC9592223

[ref22] Sakai K.; Ozawa H. Homogeneous Catalysis of Platinum(II) Complexes in Photochemical Hydrogen Production from Water. Coord. Chem. Rev. 2007, 251, 2753–2766. 10.1016/j.ccr.2007.08.014.

[ref23] Bae E.; Choi W. Effect of the Anchoring Group (Carboxylate vs Phosphonate) in Ru-Complex-Sensitized TiO_2_ on Hydrogen Production under Visible Light. J. Phys. Chem. B 2006, 110, 14792–14799. 10.1021/jp062540+. 16869588

[ref24] Förster C.; Heinze K. Photophysics and Photochemistry with Earth-Abundant Metals-Fundamentals and Concepts. Chem. Soc. Rev. 2020, 49, 1057–1070. 10.1039/C9CS00573K. 32025671

[ref25] Ran J.; Zhang J.; Yu J.; Jaroniec M.; Qiao S. Z. Earth-Abundant Cocatalysts for Semiconductor-Based Photocatalytic Water Splitting. Chem. Soc. Rev. 2014, 43, 7787–7812. 10.1039/C3CS60425J. 24429542

[ref26] Luo Y.; Li X.; Cai X.; Zou X.; Kang F.; Cheng H. M.; Liu B. Two-Dimensional MoS_2_ Confined Co(OH)_2_ Electrocatalysts for Hydrogen Evolution in Alkaline Electrolytes. ACS Nano 2018, 12, 4565–4573. 10.1021/acsnano.8b00942. 29688695

[ref27] Wender H.; Gonçalves R. V.; Dias C. S. B.; Zapata M. J. M.; Zagonel L. F.; Mendonça E. C.; Teixeira S. R.; Garcia F. Photocatalytic Hydrogen Production of Co(OH)_2_ Nanoparticle-Coated α-Fe_2_O_3_ Nanorings. Nanoscale 2013, 5, 9310–9316. 10.1039/c3nr02195e. 23948808

[ref28] Zhang L. J.; Zheng R.; Li S.; Liu B. K.; Wang D. J.; Wang L. L.; Xie T. F. Enhanced Photocatalytic H2 Generation on Cadmium Sulfide Nanorods with Cobalt Hydroxide as Cocatalyst and Insights into Their Photogenerated Charge Transfer Properties. ACS Appl. Mater. Interfaces 2014, 6, 13406–13412. 10.1021/am501216b. 25105856

[ref29] Wang Z.; Liu Y.; Gao C.; Jiang H.; Zhang J. A Porous Co(OH)_2_ Material Derived from a MOF Template and Its Superior Energy Storage Performance for Supercapacitors. J. Mater. Chem. A 2015, 3, 20658–20663. 10.1039/C5TA04663G.

[ref30] Yang J.; Zhang X.; Xu Z.; Huang J.; Chen J. Synthesis of Mesoporous Co(OH)_2_ Nanocubes Derived from Prussian Blue Analogue and Their Electrocapacitive Properties. J. Electroanal. Chem. 2017, 788, 54–60. 10.1016/j.jelechem.2017.01.045.

[ref31] Sahoo D. P.; Nayak S.; Reddy K. H.; Martha S.; Parida K. Fabrication of a Co(OH)_2_/ZnCr LDH “p-n” Heterojunction Photocatalyst with Enhanced Separation of Charge Carriers for Efficient Visible-Light-Driven H_2_ and O_2_ Evolution. Inorg. Chem. 2018, 57, 3840–3854. 10.1021/acs.inorgchem.7b03213. 29528221

[ref32] Li Z.; Wu Y.; Lu G. Highly Efficient Hydrogen Evolution over Co(OH)_2_ Nanoparticles Modified g-C_3_N_4_ Co-Sensitized by Eosin Y and Rose Bengal under Visible Light Irradiation. Appl. Catal., B 2016, 188, 56–64. 10.1016/j.apcatb.2016.01.057.

[ref33] Sun X.; Ding Y.; Zhang B.; Huang R.; Su D. S. New Insights into the Oxidative Dehydrogenation of Propane on Borate-Modified Nanodiamond. Chem. Commun. 2015, 51, 9145–9148. 10.1039/C5CC00588D. 25948395

[ref34] Zhou X.; Jin J.; Zhu X.; Huang J.; Yu J.; Wong W. Y.; Wong W. K. New Co(OH)_2_/CdS Nanowires for Efficient Visible Light Photocatalytic Hydrogen Production. J. Mater. Chem. A 2016, 4, 5282–5287. 10.1039/C6TA00325G.

[ref35] Indra A.; Paik U.; Song T. Boosting Electrochemical Water Oxidation with Metal Hydroxide Carbonate Templated Prussian Blue Analogues. Angew. Chem., Int. Ed. 2018, 57, 1241–1245. 10.1002/anie.201710809. 29214722

[ref36] Indra A.; Menezes P. W.; Das C.; Göbel C.; Tallarida M.; Schmeiβer D.; Driess M. A Facile Corrosion Approach to the Synthesis of Highly Active CoO_x_ Water Oxidation Catalysts. J. Mater. Chem. A 2017, 5, 5171–5177. 10.1039/C6TA10650A.

[ref37] Kurmoo M.; Kepert C. J. Hard Magnets Based on Transition Metal Complexes with the Dicyanamide Anion, {N(CN)_2_}^−^. New J. Chem. 1998, 22, 1515–1524. 10.1039/a803165g.

[ref38] Batten S. R.; Murray K. S. Structure and Magnetism of Coordination Polymers Containing Dicyanamide and Tricyanomethanide. Coord. Chem. Rev. 2003, 246, 103–130. 10.1016/S0010-8545(03)00119-X.

[ref39] Batten S. R.; Jensen P.; Kepert C. J.; Kurmoo M.; Moubaraki B.; Murray S.; Price D. J. Syntheses, Structures and Magnetism of α-Mn(Dca)_2_, [Mn(Dca)_2_(H_2_O)_2_].H_2_O, [Mn(Dca)_2_(C_2_H_5_OH)_2_].(CH_3_)2CO, [Fe(Dca)_2_(CH_3_OH)_2_] and [Mn(Dca)_2_(L)_2_], Where L = Pyridine, CH_3_OH or DMF and Dca^–^ = Dicyanamide, N(CN)_2_^–^. J. Chem. Soc., Dalton Trans. 1999, (17), 2987–2997. 10.1039/a903487k.

[ref40] Manson J. L.; Kmety C. R.; Huang Q. Z.; Lynn J. W.; Bendele G. M.; Pagola S.; Stephens P. W.; Liable-Sands L. M.; Rheingold A. L.; Epstein A. J.; Miller J. S. Structure and Magnetic Ordering of MII[N(CN)_2_]_2_ (M = Co, Ni). Chem. Mater. 1998, 10, 2552–2560. 10.1021/cm980321y.

[ref41] Sadigh Akbari S.; Karadas F. Selective Photocatalytic CO_2_ Reduction by Cobalt Dicyanamide. Dalton Trans. 2022, 51, 12569–12575. 10.1039/d2dt01606k. 35920585

[ref42] Batten S. R.; Robson R.; Batten S. R.; Jensen P.; Moubaraki B.; Murray K. S. Structure and Molecular Magnetism of the Rutile-Related Compounds M(Dca)_2_, Where M = CoII, NiII, CuII, Dca = Dicyanamide, N(CN)_2_^–^. Chem. Commun. 1998, 439–440. 10.1039/a707264c.

[ref43] Palion-Gazda J.; Klemens T.; Machura B.; Vallejo J.; Lloret F.; Julve M. Single Ion Magnet Behaviour in a Two-Dimensional Network of Dicyanamide-Bridged Cobalt(Ii) Ions. Dalton Trans. 2015, 44, 2989–2992. 10.1039/C4DT03574G. 25608601

[ref44] Du M.; Zhao X. J.; Batten S. R.; Ribas J. From 1-D Coordination Polymers to 3-D Hydrogen-Bonding Networks: Crystal Engineering and Magnetism of CuII-Dca-Cyanopyridine Supramolecular Systems (Dca = Dicyanamide, N(CN)_2_^–^). Cryst. Growth Des. 2005, 5, 901–909. 10.1021/cg0496888.

[ref45] Palion-Gazda J.; Choroba K.; Machura B.; Świtlicka A.; Kruszynski R.; Cano J.; Lloret F.; Julve M. Influence of the Pyrazine Substituent on the Structure and Magnetic Properties of Dicyanamide-Bridged Cobalt(II) Complexes. Dalton Trans. 2019, 48, 17266–17280. 10.1039/C9DT02976A. 31713552

[ref46] Mann M.; Mroz D.; Henrich L.; Houben A.; Van Leusen J.; Dronskowski R. Syntheses and Characterization of Diammine-Nickel/Cobalt(II)-Bisdicyanamide M(NH_3_)_2_[N(CN)_2_]_2_ with M = Ni and Co. Inorg. Chem. 2019, 58, 7803–7811. 10.1021/acs.inorgchem.9b00448. 31185550

[ref47] Sivanantham A.; Ganesan P.; Estevez L.; McGrail B. P.; Motkuri R. K.; Shanmugam S. A Stable Graphitic, Nanocarbon-Encapsulated, Cobalt-Rich Core-Shell Electrocatalyst as an Oxygen Electrode in a Water Electrolyzer. Adv. Energy Mater. 2018, 8, 1702838 10.1002/aenm.201702838.

[ref48] Hellgren N.; Haasch R. T.; Schmidt S.; Hultman L.; Petrov I. Interpretation of X-Ray Photoelectron Spectra of Carbon-Nitride Thin Films: New Insights from in Situ XPS. Carbon 2016, 108, 242–252. 10.1016/j.carbon.2016.07.017.

[ref49] Chang J. K.; Lee M. T.; Tsai W. T.; Deng M. J.; Sun I. W. X-Ray Photoelectron Spectroscopy and in Situ X-Ray Absorption Spectroscopy Studies on Reversible Insertion/Desertion of Dicyanamide Anions into/from Manganese Oxide in Ionic Liquid. Chem. Mater. 2009, 21, 2688–2695. 10.1021/cm9000569.

[ref50] Hashimoto H.; Ohno A.; Nakajima K.; Suzuki M.; Tsuji H.; Kimura K. Surface Characterization of Imidazolium Ionic Liquids by High-Resolution Rutherford Backscattering Spectroscopy and X-Ray Photoelectron Spectroscopy. Surf. Sci. 2010, 604, 464–469. 10.1016/j.susc.2009.12.023.

[ref51] Ivanova T.; Naumkin A.; Sidorov A.; Eremenko I.; Kiskin M. X-Ray Photoelectron Spectra and Electron Structure of Polynuclear Cobalt Complexes. J. Electron Spectrosc. Relat. Phenom. 2007, 156–158, 200–203. 10.1016/j.elspec.2006.12.005.

[ref52] Haraguchi H.; Fujiwara K.; Fuwa K. A Study of Cobalt Complexes by X-Ray Photoelectron Spectroscopy. Chem. Lett. 1975, 4, 409–414. 10.1246/cl.1975.409.

[ref53] Briggs D.; Gibson V. A. Direct Observation of Multiplet Splitting in 2P Photoelectron Peaks of Cobalt Complexes. Chem. Phys. Lett. 1974, 25, 493–496. 10.1016/0009-2614(74)85350-9.

[ref54] Vernon G. A.; Stucky G.; Carlson T. A. Comprehensive Study of Satellite Structure in the Photoelectron Spectra of Transition Metal Compounds. Inorg. Chem. 1976, 15, 278–284. 10.1021/ic50156a007.

[ref55] Damrauer N. H.; Cerullo G.; Yeh A.; Boussie T. R.; Shank C. V.; McCusker J. K. Femtosecond Dynamics of Excited-State Evolution in [Ru(bpy)_3_]^2+^. Science 1997, 275, 54–57. 10.1126/science.275.5296.54. 8974388

[ref56] Beyene B. B.; Hung C. H. Photocatalytic Hydrogen Evolution from Neutral Aqueous Solution by a Water-Soluble Cobalt(II) Porphyrin. Sustainable Energy Fuels 2018, 2, 2036–2043. 10.1039/C8SE00253C.

[ref57] Joliat-Wick E.; Weder N.; Klose D.; Bachmann C.; Spingler B.; Probst B.; Alberto R. Light-Induced H_2_ Evolution with a Macrocyclic Cobalt Diketo-Pyrphyrin as a Proton-Reducing Catalyst. Inorg. Chem. 2018, 57, 1651–1655. 10.1021/acs.inorgchem.7b02992. 29368926

[ref58] Sang X.; Zhang J.; Wu T.; Zhang B.; Ma X.; Peng L.; Han B.; Kang X.; Liu C.; Yang G. Room-Temperature Synthesis of Mesoporous CuO and Its Catalytic Activity for Cyclohexene Oxidation. RSC Adv. 2015, 5, 67168–67174. 10.1039/C5RA12808K.

[ref59] Singh M.; Jampaiah D.; Kandjani A. E.; Sabri Y. M.; Della Gaspera E.; Reineck P.; Judd M.; Langley J.; Cox N.; Van Embden J.; Mayes E. L. H.; Gibson B. C.; Bhargava S. K.; Ramanathan R.; Bansal V. Oxygen-Deficient Photostable Cu_2_O for Enhanced Visible Light Photocatalytic Activity. Nanoscale 2018, 10, 6039–6050. 10.1039/C7NR08388B. 29543296

[ref60] Chaki N. K.; Sudrik S. G.; Sonawane H. R.; Vijayamohanan K. Single Phase Preparation of Monodispersed Silver Nanoclusters Using a Unique Electron Transfer and Cluster Stabilising Agent, Triethylamine. Chem. Commun. 2002, 2, 76–77. 10.1039/b107965b. 12120318

[ref61] De Silva O.; Singh M.; Mahasivam S.; Mahmood N.; Murdoch B. J.; Ramanathan R.; Bansal V. Importance of Phase Purity in Two-Dimensional β-Co(OH)_2_ for Driving Oxygen Evolution. ACS Appl. Nano Mater. 2022, 5, 12209–12216. 10.1021/acsanm.2c03468.

[ref62] Klissurski D. G.; Uzunova E. L. Synthesis of Nickel Cobaltite Spinel from Coprecipitated Nickel-Cobalt Hydroxide Carbonate. Chem. Mater. 1991, 3, 1060–1063. 10.1021/cm00018a021.

[ref63] Xu R.; Zeng H. C. Dimensional Control of Cobalt-Hydroxide-Carbonate Nanorods and Their Thermal Conversion to One-Dimensional Arrays of Co_3_O_4_ Nanoparticles. J. Phys. Chem. B 2003, 107, 12643–12649. 10.1021/jp035751c.

[ref64] Liu Z.; Ma R.; Osada M.; Takada K.; Sasaki T. Selective and Controlled Synthesis of α- and β-Cobalt Hydroxides in Highly Developed Hexagonal Platelets. J. Am. Chem. Soc. 2005, 127, 13869–13874. 10.1021/ja0523338. 16201808

[ref65] Li H. B.; Yu M. H.; Lu X. H.; Liu P.; Liang Y.; Xiao J.; Tong Y. X.; Yang G. W. Amorphous Cobalt Hydroxide with Superior Pseudocapacitive Performance. ACS Appl. Mater. Interfaces 2014, 6, 745–749. 10.1021/am404769z. 24386890

[ref66] Xu Z. P.; Zeng H. C. Interconversion of Brucite-like and Hydrotalcite-like Phases in Cobalt Hydroxide Compounds. Chem. Mater. 1999, 11, 67–74. 10.1021/cm980420b.

[ref67] Li X.; Hou J.; Sui H.; Sun L.; Xu L. Switchable-Hydrophilicity Triethylamine: Formation and Synergistic Effects of Asphaltenes in Stabilizing Emulsions Droplets. Materials 2018, 11, 2431 10.3390/ma11122431. 30513618 PMC6316943

[ref68] Liang Z.; Zhang C.; Xu Y.; Zhang W.; Zheng H.; Cao R. Dual Tuning of Ultrathin α-Co(OH)_2_ Nanosheets by Solvent Engineering and Coordination Competition for Efficient Oxygen Evolution. ACS Sustain. Chem. Eng. 2019, 7, 3527–3535. 10.1021/acssuschemeng.8b05770.

