# Supplementary material for: 3D-Cobalt-Dicyanamide-Derived 2D-Layered-Co(OH)2-Based Catalyst for Light-Driven Hydrogen Evolution
Source: ACS Omega. 2024 Feb 9;9(7):8585–93. doi: 10.1021/acsomega.4c00217 (PMC10883017; doi:10.1021/acsomega.4c00217)
Supplement: Supplementary file 1 — ao4c00217_si_001.pdf [file ao4c00217_si_001.pdf]

Supporting Information

A 3D Cobalt Dicyanamide-Derived 2D-Layered $Co(OH)_2$-Based Catalyst for Light-Driven Hydrogen

Sina Sadigh Akbari,[1,ǂ] *Ferdi Karadas*[1,2,*]

[1] *Department of Chemistry, Faculty of Science, Bilkent University, 06800 Ankara, Turkey.*

[2] *UNAM - National Nanotechnology Research Center, Institute of Materials Science and Nanotechnology, Bilkent University, 06800 Ankara, Turkey.*

[ǂ] *Present address: Department of Chemistry, Johannes Gutenberg University Mainz, 55128 Mainz, Germany.*

* Corresponding author: Ferdi Karadas, Email: karadas@fen.bilkent.edu.tr.

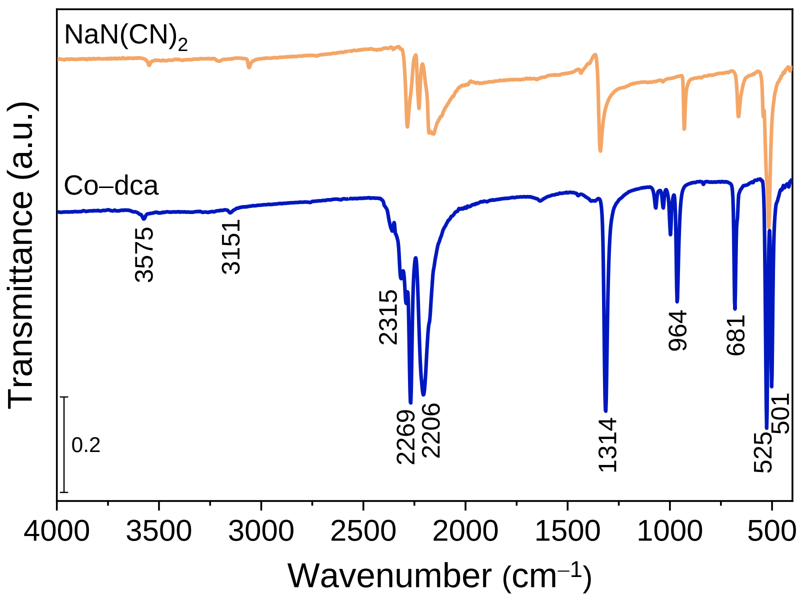

Figure S1. ATR–FTIR spectra of Co–dca sample and $NaN(CN)_2$.

Table S1. ATR–FTIR data of Co–dca sample and $NaN(CN)_2$.

Vibration	$NaN(CN)_2$ wavenumber (cm^{-1})	Co–dca wavenumber (cm^{-1})
**δ(N−C≡N)**	516	501
**γ(N−C≡N)**	542	525
**(N−C≡N)**	663	681
ν_s**(N−C)**	929	964
ν_{as}**(N−C)**	1340	1314
ν_s**(C≡N)**	2176	2206
ν_{as}**(C≡N)**	2227	2269
$\nu_{as} + \nu_s$**(C≡N)**	2284	2315
ν_{as}**(C≡N) +** ν_s**(N−C)**	3058	3151
ν_s**(C≡N) +** ν_{as}**(N−C)**	3548	3575

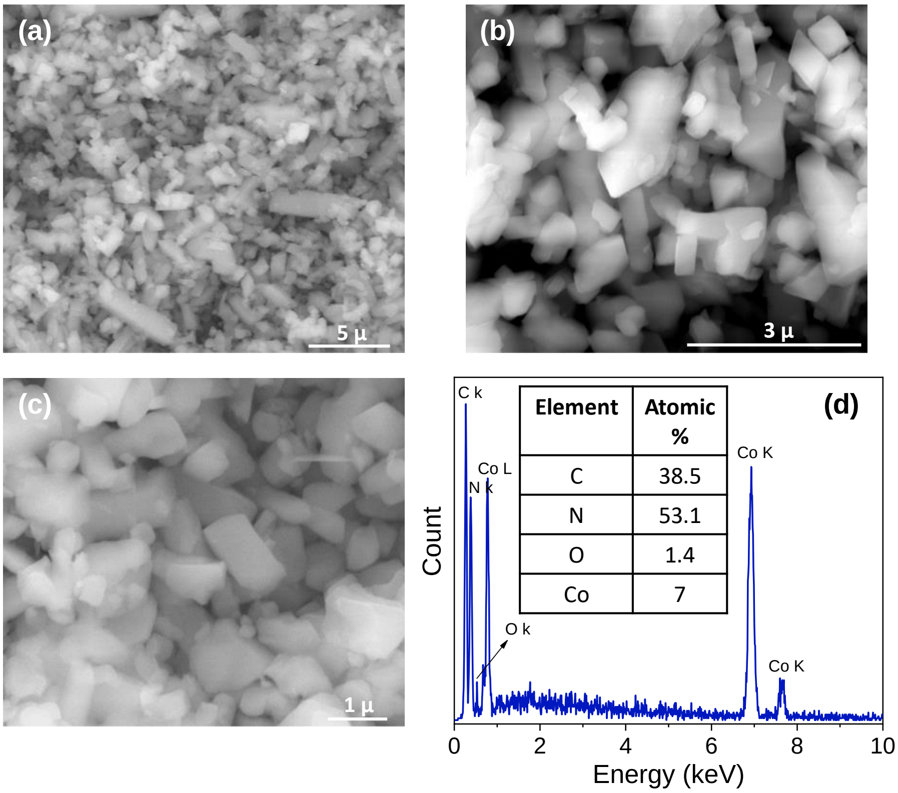

Figure S2. (a–c) SEM images, and (d) SEM–EDS analysis of Co–dca. Inset: The atomic ratio of all elements for the Co–dca.

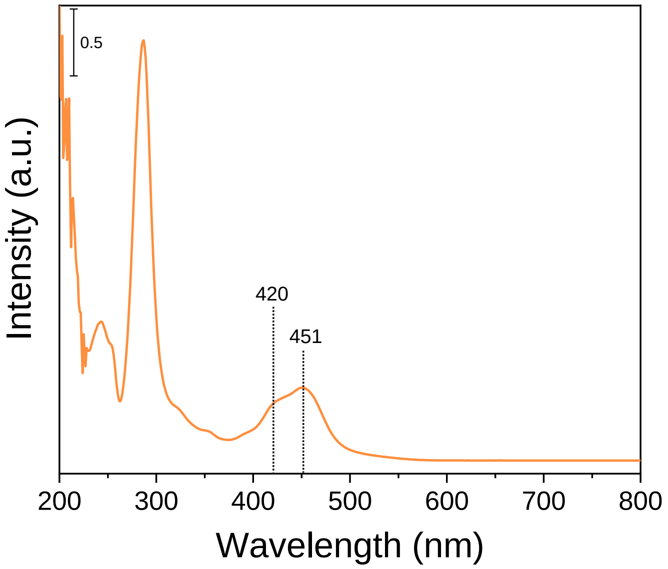

Figure S3. UV–Vis absorption spectrum of $[Ru(bpy)_3](PF_6)_2$ in $MeCN/H_2O$ solution.

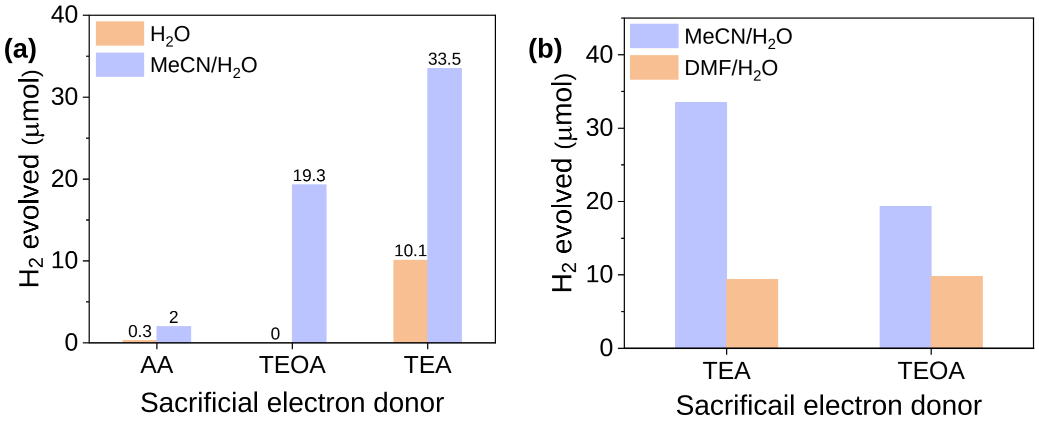

Figure S4. H_2 production activity of Co–dca (a) in the MeCN/H_2O (19/1, v/v) mixed solution (purple bars) and 100% (volume %) water (orange bars) in the presence of triethylamine (TEA), triethanolamine (TEOA), and ascorbic acid (AA) as the sacrificial electron donors, and (b) in the MeCN/H_2O (purple bars) and DMF/H_2O (orange bars) mixed solutions in the presence of TEA and TEOA as the electron donor reagents. The reactions are performed using 1 mM [Ru(bpy)$_3$](PF$_6$)$_2$ and 10 mg of Co–dca under visible light irradiation (λ > 420 nm) for 2 h.

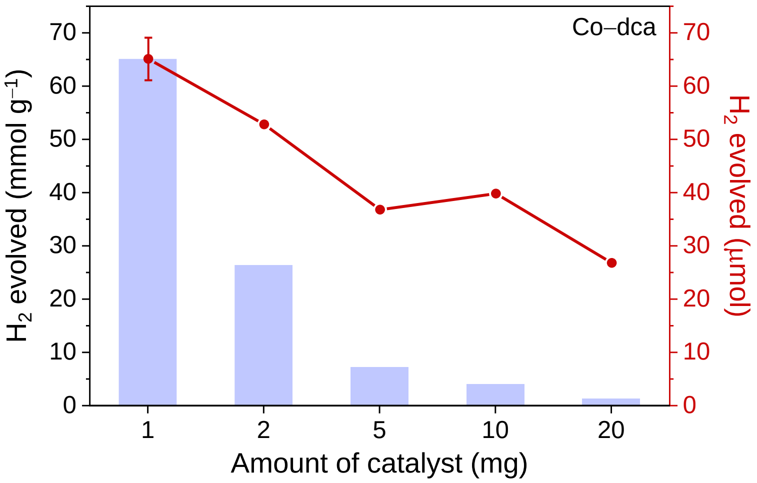

Figure S5. The effect of the quantity of Co–dca on the H_2 yield in 8 mL MeCN/H_2O (19/1, v/v) solution with 1 mM of [Ru(bpy)$_3$](PF$_6$)$_2$, and 2 mL of TEA under visible light irradiation (λ > 420 nm) for 3 h.

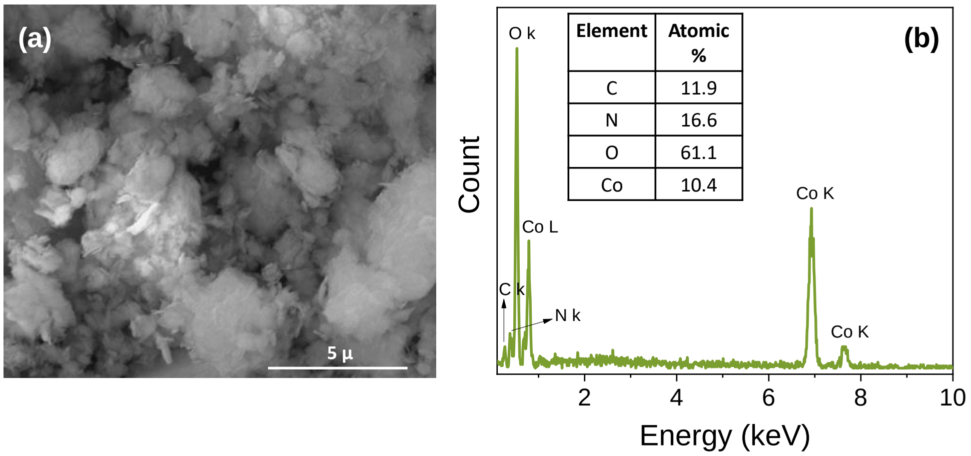

Figure S6. (a) SEM image, and (b) SEM–EDS analysis of Co–dca/TEA. Inset: The atomic ratio of all elements for the Co–dca/TEA.

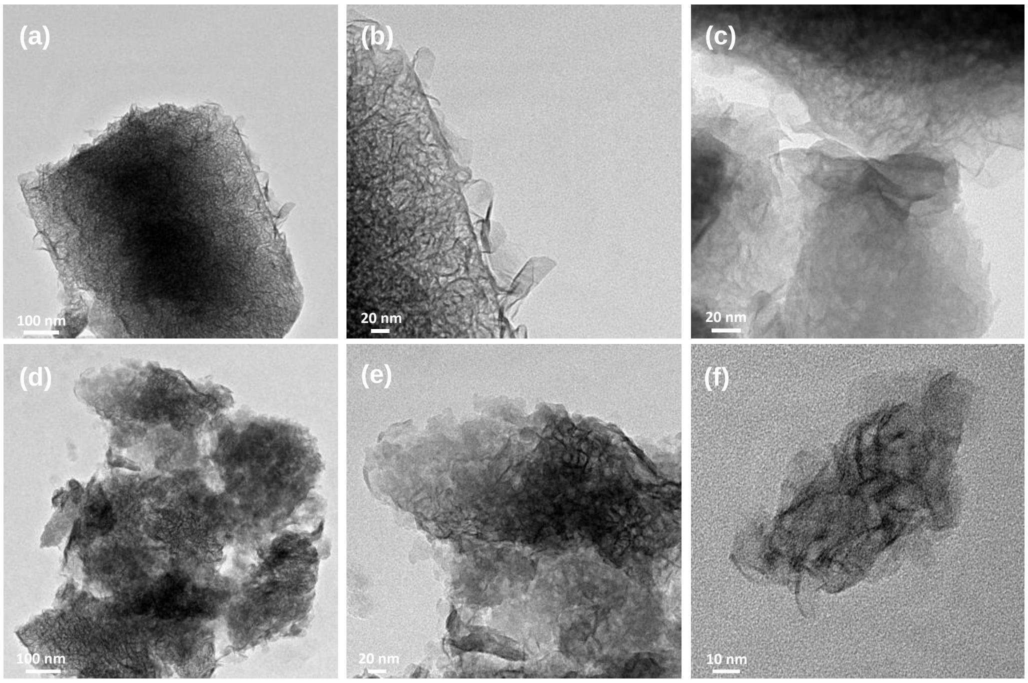

Figure S7. (a–f) TEM images of Co–dca/TEA.

Table S2. Comparison study between catalysts in this work and previously reported photocatalysts for HER.

**Photocatalyst**	**Incident light (nm)**	**Sacrificial agent**	**H_2 evolved (μmol g^{-1} h^{-1})**	**Ref.**
**$Fe_2O_3/Co(OH)_2$**	UV–Vis	Ethanol	140	1
**$CdS/Co(OH)_2$**	UV–Vis	Ethanol	61	2
**$TiO_2/Co(OH)_2$**	UV–Vis	Methanol	1946	3
**$CdS/Co(OH)_2$**	> 420	TEOA	14430	4
**$TiO_2/Co(OH)_2$**	UV–Vis	Methanol	746	5
**ZnCr-LDH/$Co(OH)_2$**	> 420	Methanol	27878	6
**$[Ru(bpy)_3]^{2-}$/Co–dca**	> 420	TEA	27900	This Work
**$[Ru(bpy)_3]^{2-}$/Co–dca/TEA**	> 420	TEA	28300	This work

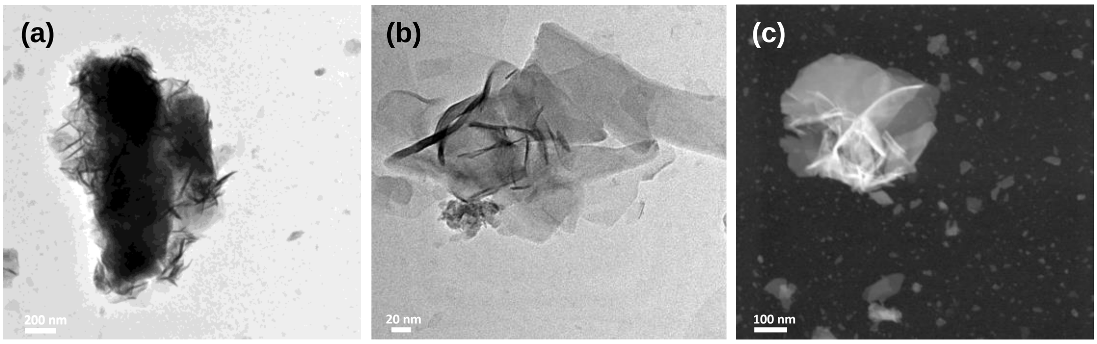

Figure S8. (a–b) TEM images, and (c) HAADF–STEM of post-catalytic Co–dca.

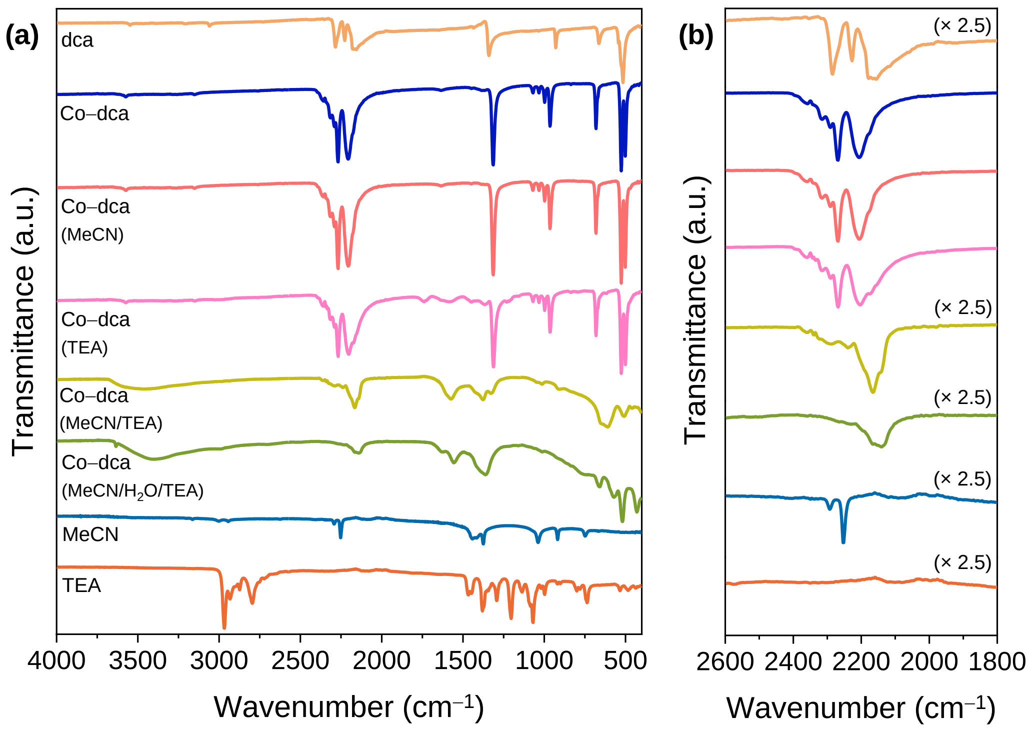

Figure S9. ATR–FTIR spectra of Co–dca dispersed in different solutions (a) 400–4000 cm^{-1}, and (b) 1800–2600 cm^{-1}. A 10 mg of Co–dca powder was dispersed in 10 mL of solution, and stirred for 3 h at room temperature. Then, the solid was centrifuged, and dried at 75 °C overnight.

References:

(1) Wender, H.; Gonçalves, R. V.; Dias, C. S. B.; Zapata, M. J. M.; Zagonel, L. F.; Mendonça, E. C.; Teixeira, S. R.; Garcia, F. Photocatalytic Hydrogen Production of $Co(OH)_2$ Nanoparticle-Coated α-Fe_2O_3 Nanorings. *Nanoscale* **2013**, *5*, 9310–9316.

(2) Zhang, L. J.; Zheng, R.; Li, S.; Liu, B. K.; Wang, D. J.; Wang, L. L.; Xie, T. F. Enhanced Photocatalytic H_2 Generation on Cadmium Sulfide Nanorods with Cobalt Hydroxide as Cocatalyst and Insights into Their Photogenerated Charge Transfer Properties. *ACS Appl. Mater. Interfaces* **2014**, *6*, 13406–13412.

(3) Dang, H.; Dong, X.; Dong, Y.; Fan, H.; Qiu, Y. Facile Synthesis of $Co(OH)_2$ Modified TiO_2 Nanocomposites with Enhanced Photocatalytic H_2 Evolution Activity. *Mater. Lett.* **2015**, *138*, 56–59.

(4) Zhou, X.; Jin, J.; Zhu, X.; Huang, J.; Yu, J.; Wong, W. Y.; Wong, W. K. New $Co(OH)_2$/CdS Nanowires for Efficient Visible Light Photocatalytic Hydrogen Production. *J. Mater. Chem. A* **2016**, *4*, 5282–5287.

(5) Xu, H.; Li, S.; Ge, L.; Han, C.; Gao, Y. In-Situ Synthesis of Novel Plate-like $Co(OH)_2$ Co-Catalyst Decorated TiO_2 Nanosheets with Efficient Photocatalytic H_2 Evolution Activity. *Int. J. Hydrogen Energy* **2017**, *42*, 22877–22886.

(6) Sahoo, D. P.; Nayak, S.; Reddy, K. H.; Martha, S.; Parida, K. Fabrication of a $Co(OH)_2$/ZnCr LDH "p-n" Heterojunction Photocatalyst with Enhanced Separation of Charge Carriers for Efficient Visible-Light-Driven H_2 and O_2 Evolution. *Inorg. Chem.* **2018**, *57*, 3840–3854.
